# A Fast Healthcare Interoperability Resources (FHIR) layer implemented over i2b2

**DOI:** 10.1186/s12911-017-0513-6

**Published:** 2017-08-14

**Authors:** Abdelali Boussadi, Eric Zapletal

**Affiliations:** 1 INSERM UMR 1138, Equipe 22, Centre de Recherche des Cordeliers, Universités Paris 5 et 6, Paris, France; 2 Département de Santé Publique et Informatique Médicale, Hôpital Européen Georges Pompidou, Assistance Publique – Hôpitaux de Paris, Paris, France

**Keywords:** FHIR, Semantic interoperability, Database management systems, Clinical data warehouse, i2b2

## Abstract

**Background:**

Standards and technical specifications have been developed to define how the information contained in Electronic Health Records (EHRs) should be structured, semantically described, and communicated. Current trends rely on differentiating the representation of data instances from the definition of clinical information models. The dual model approach, which combines a reference model (RM) and a clinical information model (CIM), sets in practice this software design pattern. The most recent initiative, proposed by HL7, is called Fast Health Interoperability Resources (FHIR). The aim of our study was to investigate the feasibility of applying the FHIR standard to modeling and exposing EHR data of the Georges Pompidou European Hospital (HEGP) integrating biology and the bedside (i2b2) clinical data warehouse (CDW).

**Results:**

We implemented a FHIR server over i2b2 to expose EHR data in relation with five FHIR resources: DiagnosisReport, MedicationOrder, Patient, Encounter, and Medication. The architecture of the server combines a Data Access Object design pattern and FHIR resource providers, implemented using the Java HAPI FHIR API. Two types of queries were tested: query type #1 requests the server to display DiagnosticReport resources, for which the diagnosis code is equal to a given ICD-10 code. A total of 80 DiagnosticReport resources, corresponding to 36 patients, were displayed. Query type #2, requests the server to display MedicationOrder, for which the FHIR Medication identification code is equal to a given code expressed in a French coding system. A total of 503 MedicationOrder resources, corresponding to 290 patients, were displayed. Results were validated by manually comparing the results of each request to the results displayed by an ad-hoc SQL query.

**Conclusion:**

We showed the feasibility of implementing a Java layer over the i2b2 database model to expose data of the CDW as a set of FHIR resources. An important part of this work was the structural and semantic mapping between the i2b2 model and the FHIR RM. To accomplish this, developers must manually browse the specifications of the FHIR standard. Our source code is freely available and can be adapted for use in other i2b2 sites.

## Introduction

The wide adoption of electronic health records (EHRs) [1] has made a large amount of data available to various actors, including executives, physicians, researchers, etc., for various purposes, including administration, management, clinical practice, and research [2]. Many groups have promoted the secondary use of EHR data for clinical research [3, 4]. Reusing EHR data can aid clinical research by reducing redundant data capture, providing a better understanding of real patient populations, testing hypotheses, verifying clinical trial feasibility, screening populations, recruiting patients, detecting safety risks early, analyzing treatment outcomes, and supporting post-marketing monitoring and long-term surveillance [5]. Deriving benefits from the reuse of EHR data through data warehousing appears to be the best strategy [6], because operational databases relying on EHRs are difficult to query given the multiplicity of relational tables, which are optimized to facilitate patient management, but are not suitable for research purposes [7]. In addition, querying operational systems can be computationally intensive and requires specific local expertise [8]. The Informatics for Integrating Biology and the Bedside (i2b2) project is the most widely used clinical data warehouse in the world [9]. I2b2 has already been used to data warehouse EHR data across multiple institutions in a large European project (EHR4CR) which aimed to demonstrate a scalable, widely acceptable, and efficient approach to interoperability between EHR and clinical research systems [10].

In the EHR4CR project, the authors showed that one of the most important barriers to reach the objective of reusing EHR data across different institutions was the non-interoperability of their underlying storage systems, regardless of the chosen strategy [10]. Interoperability of EHRs has become an important objective for several countries [11] and various international projects have been funded to develop, assess, and enhance the use of interoperability semantic standards to share EHR data between different institutions [10, 12].

Standards and technical specifications have been developed by various international organizations to define how the information contained in EHRs should be structured, semantically described, and communicated [13]. Indeed, current trends followed by most of these organizations rely on differentiating the representation of data instances from the definition of clinical information models. Structuring of data should be handled by “a syntactic (or technical) interoperability layer” whereas data semantics should be handled by “a semantic interoperability layer”. Communication is handled by both, as well as a third interoperability layer called the “process interoperability layer” [14].

The dual model approach [15], which combines a reference model (RM) and a clinical information model (CIM), implements this software design pattern. The RM is a high-level abstraction model composed of a set of generic classes, which should be included in any EHR, to structure clinical data (syntactic interoperability). The CIM is a terminology-ontology-constrained subset of the RM which allows semantic enrichment of the RM. The clinical artifacts resulting from this design process will be easily organized, stored, queried, displayed, analyzed for various purposes, and exchanged between different information systems [13].

Goosen et al. [16] has described the most popular initiatives that follow the dual model approach, highlighting their differences and similarities. Most have been assessed in various projects to expose EHR data: Chen et al. [17] used the openEHR Archetype to convert data of a regional EHR system and Spath et al. [18] to model data of a biobank database. Goosen et al. [19] used the HL7 (Health Level Seven International) RIM to represent nursing content and Dufschmid et al. [14] to share EHR data, using the ISO/EN 13606 Archetypes. The most recent initiative, proposed by HL7, is called Fast Health Interoperability Resources (FHIR, pronounced “Fire”), which is a new generation specification that uses modular components called “resources”.

FHIR have received increasing attention as a content definition standard by the Harvard SMART project [20] and other wide public initiative’s/public initiative’s [21, 22] since the first specification drafts were adopted. Few articles have investigated the issue of exposing i2b2 data using FHIR. The only study to investigate this issue is the work described by Wagholikar et al. [23]. The authors built an i2b2 plugin based on the SMART-on-FHIR profile [20]. The primary aim of this plugin was to run SMART-on-FHIR-based mobile applications on an i2b2 data warehouse. It can also be used to expose i2b2 data in the FHIR format for a FHIR-client. This plugin is limited in scope, serving data on a per patient basis [23].

The aim of our study was to investigate the feasibility of applying the HL7 FHIR standard to modeling and exposing EHR data of the Georges Pompidou European Hospital (HEGP) i2b2 clinical data warehouse (CDW). Our approach is an alternative to that described by Wagholikar et al. [23]. It is based on a locally developed FHIR profile and a purely Java API for FHIR called HAPI FHIR [24] and will not be limited to serve data on a per patient basis.

## Background

### The HEGP i2b2 clinical data warehouse

HEGP is a teaching hospital with 74 clinical departments and 827 beds. The HEGP clinical information system integrates an electronic health record with a Computerized Prescriber Order Entry (CPOE) [25]. A CDW based on the i2b2 framework [26] was integrated into the HEGP clinical information system in 2009 [27]. The content of the HEGP CDW is described in Table 1.

**Table 1:** Content of the HEGP CDW (as of July 1st, 2016) [27]

	September 2009	December 2013	July 2016
Concepts			
Biology (thousands)	7.29	9.1	11.2
Diagnostic codes (ICD-10) (thousands)	21.39	39.91	40.25
Drugs (thousands)	31.36	33.67	41.6
Data facts			
ICD Diagnosis (millions)	1.87	2.94	7.67
Clinical items (millions)	20.8	61.1	122.2
Laboratory results (millions)	62.8	98.0	124.3
Drug orders (millions)	0.95	3.2	6.4
Text reports (millions)	0.16	2.36	3.7

The HEGP CDW contains most of the patient data produced within its clinical information system (CIS), including records of more than 606,524 different patients (since the hospital opened in July 2000): patient histories, demographics, diagnoses, symptoms, drug prescriptions and treatments, clinical and laboratory results, full-text inpatient and outpatient reports, etc. All these data are stored within the i2b2 data model, which is based on a “star schema” with a central “fact table” surrounded by several “dimension tables”. Observations concerning patients are stored within the central fact table (Observation_fact table), recorded by a specific business actor (Provider_Dimension table) within a specific time range (defined by a start date and an end date), regarding a specific concept (Concept_Dimension table), which can be a lab test or disease, a medication code, etc., in the context of an encounter (Visit_Dimension table) for a specific patient (Patient_Dimension table). Concepts in the i2b2 data model are expressed according to the entity-attribute-value (EAV) model, in which each value is stored as a row rather than as a column. Querying data using these two “design patterns” (star schema model and EAV model) is highly efficient because it allows one very large index to be built which cuts across all patient data in the fact table [26].

### Design and implementation scenario

The present work is a part of a research project for which the aim is to design, develop, and assess a full-FHIR clinical decision support system (CDSS) to control medication prescriptions in the elderly: IM-AGE (Iatrogenie Médicamenteuse et Personnes Agées).

In our scenario, the CDSS, which is currently under development, will play the role of the FHIR resources client. After interviews with the software design architect who oversees development of the CDSS, the following input-data requirements should be exposed in FHIR format:

- A set of medication ‘codes’ prescribed to elderly patients and data related to the corresponding orders: administration plan, doses, dosage, etc.
  - The patient’s birth date, the hospital patient identifier for each patient related to these medications
  - The encounter start date and end date for each medication prescription and the encounter number
- A set of diagnosis “codes” related to with elderly patients
  - The patient’s birth date, the hospital patient identifier for each patient related to these medications
  - The encounter start date and end date for each medication prescription and the encounter number

## Implementation

### Description and analysis of end user requirements

**Fig. 1:**
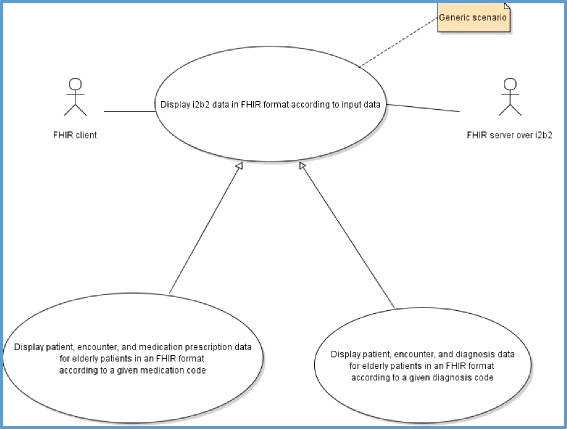
UML use case diagram describing the client requirements

We formalized his requirements using UML notation diagrams to respond to the “client” needs. Two major scenarios were selected with the “client” (Fig. 1):

1. Display patient, encounter, and medication prescription data for elderly patients in an FHIR format according to a given medication code.
2. Display patient, encounter, and diagnosis data for elderly patients in an FHIR format according to a given diagnosis code.

**Fig. 2:**
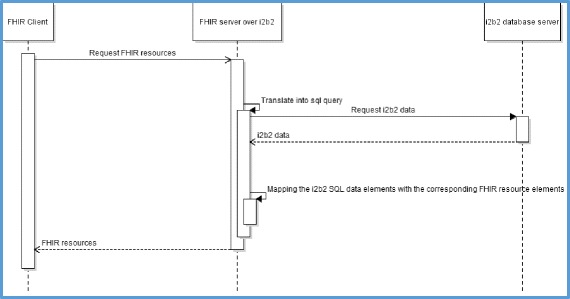
UML sequence diagram describing the execution of the generic scenario

Figure 2 shows a UML sequence diagram describing the execution of the generic scenario.

### Profiling

**Fig. 3:**
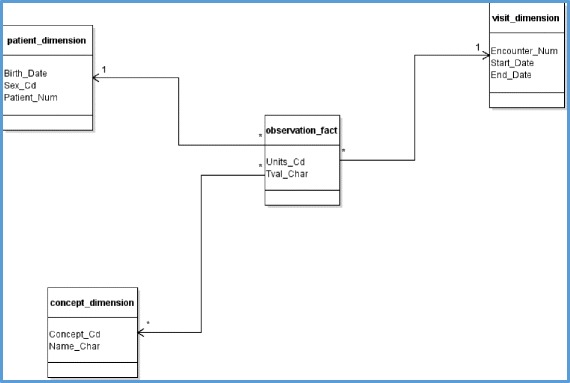
The i2b2 star schema with only the targeted data

FHIR resources are a set of information models that define data elements, constraints, and relationships for the most relevant “business objects” in the healthcare context [28]. However, resources most often need to be adapted to a local context. This mechanism is called profiling. We compared the local i2b2 data structure and storage model to the corresponding FHIR resources to determine whether it was necessary to define a profile. We first analyzed the i2b2 schema and data organization in collaboration with the HEGP i2b2 database administrator to identify the data related to the requirements of the client. These data are described in Fig. 3, in which we depict the i2b2 star schema with only the targeted entities, relations, and fields.

**Fig. 4:**
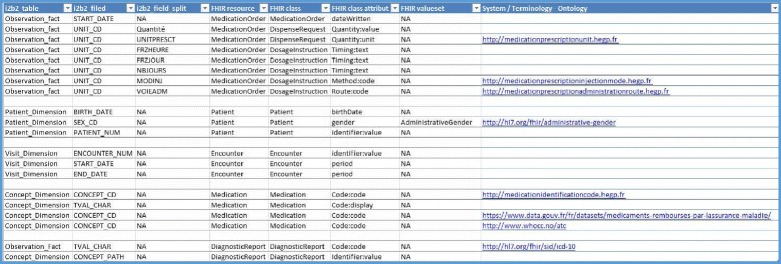
Mapping table between the i2b2 database fields and the FHIR resources field

Second, we manually browsed and analyzed the FHIR resources to identify the corresponding data elements required for the HEGP local context and adaptation, if necessary. Five FHIR resources were selected: Patient, Medication, Encounter, MedicationOrder, and DiagnosticReport. Third, we followed the method described by Lee et al. [29] and conducted a gap analysis between these two models. Figure 4 describes the mapping between these two models. We mapped all data elements from the local model to a corresponding data element in a FHIR resource and defined system terminology constraints for some to comply with the local context and ensure interoperability with external systems.

Finally, we implemented and validated the resulting mapping using the HAPI FHIR API. HAPI FHIR [24] is a Java open-source implementation of the FHIR specifications. For the work described here, we used HAPI structure library DSTU2, version 1.2, which corresponds to the FHIR specification version 1.0.0.

### The i2b2 FHIR server architecture

**Fig. 5:**
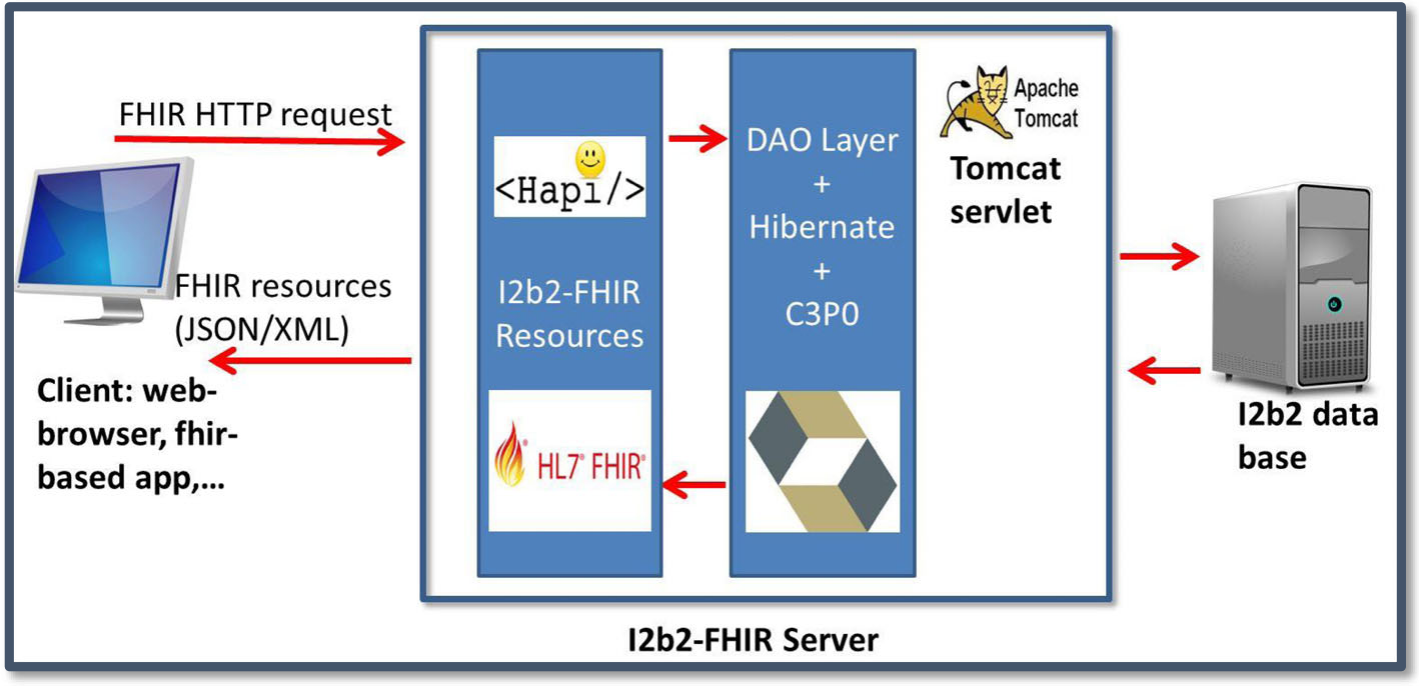
The i2b2 FHIR server architecture

The current architecture of the i2b2 FHIR server is composed of two main packages of Java classes (Fig. 5). The first, called: “i2b2 FHIR Resources,” handles the client (web browser, FHIR based app…) FHIR HTTP requests. The second, called: “DAO layer,” allows setting up of the mapping between the requested FHIR resources and the i2b2 data.

#### The i2b2-FHIR resource layer

This layer contains:

- The FHIR resource provider classes: we have defined a Java class for each FHIR resource that should be served i.e. DiagnosticReport and MedicationOrder. In these two classes, we have implemented the mapping between the local i2b2 data model and the FHIR resources described in Fig. 4. Patient, Encounter, and Medication resources have also been included in this mapping. Three coding systems and one HL7 ValueSet were used: ICD-10, ATC, and CIP codes, described later, and the administrative gender codes ValueSet. If a field was related to the use of a local code system, we identified its system with a URL, such as http://<codename > .hegp.fr (Fig. 4). If the profile is maintained and one or several FHIR resources are added to the profile, developers should add a corresponding FHIR resource provider class for each new targeted resource.
- The FHIR server class: a Java class which extends the Java HAPI FHIR RestfulServer class to initialize the servlet, defines the URL pattern for the servlet, defining the encoding format of the HTTP response (JSON/XML).

#### The data access object (DAO) layer

**Fig. 6:**
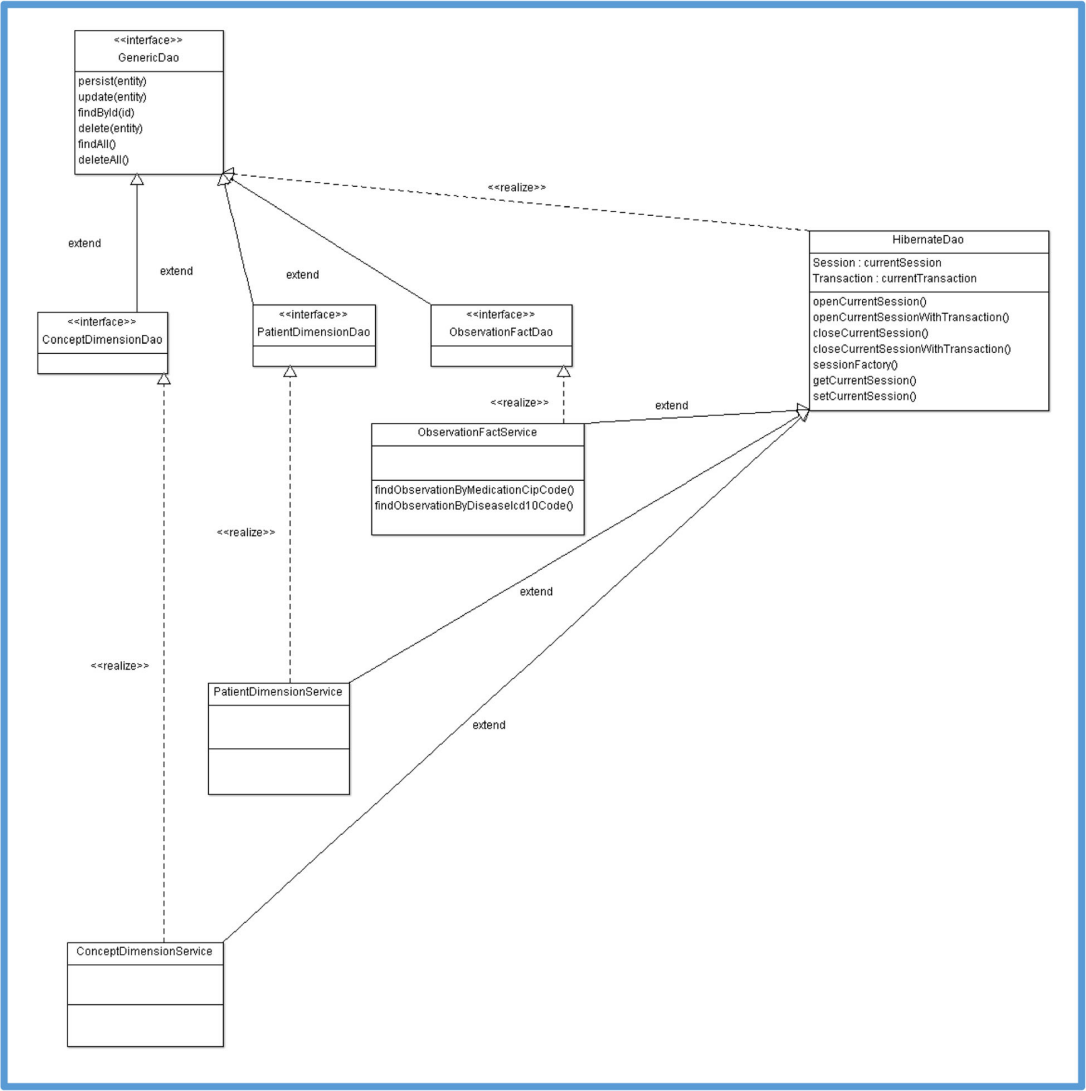
The i2b2 FHIR server DAO UML class diagram

We implemented this layer using the DAO design pattern [30] combined with a java object-relational mapping tool: Hibernate. Figure 6 describes the current UML design class diagram of the DAO Layer. A generic abstract DAO class contains the basic Create, Read, Update, and Delete (CRUD) methods; this class is extended by a set of DAO interface classes which are implemented by a set of “service” classes. The service classes contain concrete implementation of the CRUD methods. We implemented three methods in the ObservationFactService class in response to the client requirements to allow retrieval of:

- Patient data related to a given ICD-10 (International Statistical Classification of Diseases and Related Health Problems, 10th revision) code (findObservationsByDiseaseIcd10Code).
- Patient data related to a given CIP medication code prescribed to the patient (findObservationsByMedicationCipCode). CIP (Code Identifiant de Présentation) codes are widely used French identification codes for medication defined by the French national security agency for drugs and healthcare products (ANSM, Agence National de Sécurité du Médicament et des Produits de Santé) [31].
- Patient data related to a given ATC (Anatomical Therapeutic Chemical) drug code prescribed to the patient (findObservationsByMedicationCipCode).

For each of these methods, a corresponding HQL (Hibernate Query Language) query has been defined to query the i2b2 data model. Basic operations on database sessions are handled by the class HibernateDao (Fig. 6).

This layer also contains a set of Java classes called entities. Entities implement the Java representation of each i2b2 database table. For each i2b2 database table column we defined a Java class field with the same name. For each Java class field, we based the datatype mapping on the Oracle mapping table described in this reference [32].

In this layer, the entities, generic abstract DAO class, and set of DAO interface classes should not be modified except if the i2b2 star schema model is modified.

If the profile is maintained in the future and a new data element needs to be served, developers should first identify to which i2b2 table this data element belongs. They should modify the HQL query of the corresponding service class or create a new service class if it does not exist.

## Results

**Fig. 7:**
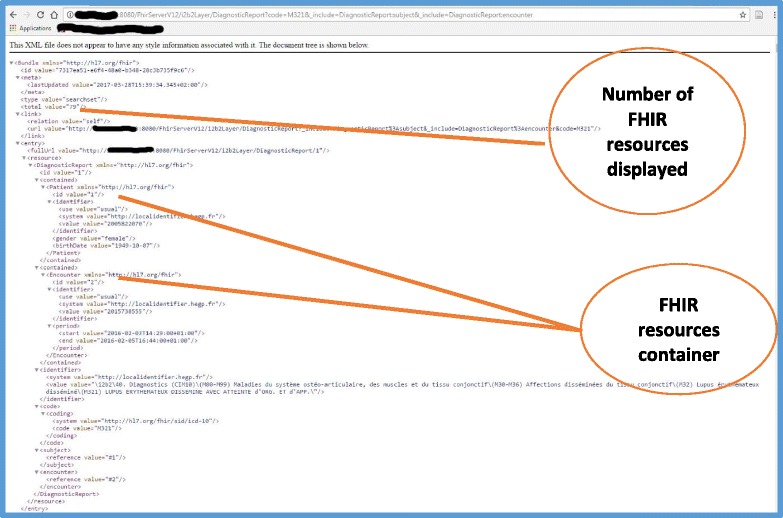
FHIR DiagnosticReport resources displayed by the server in XML format in response to query #1

**Fig. 8:**
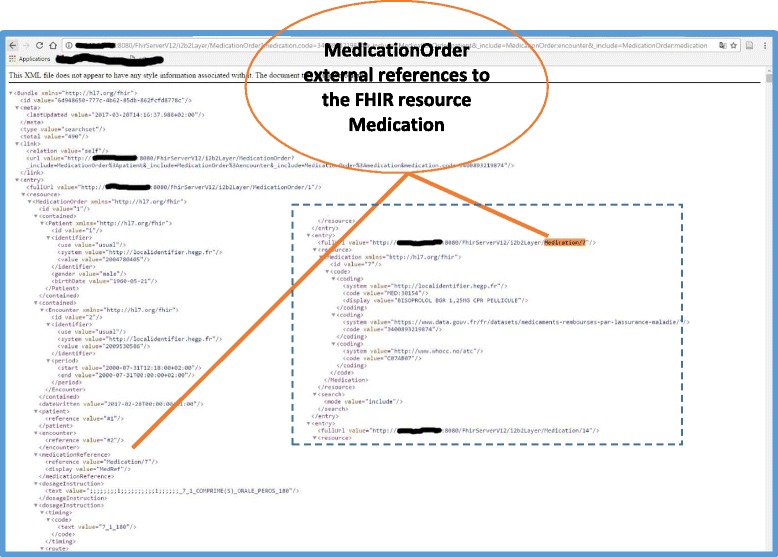
FHIR MedicationOrder resources displayed by the server in XML format in response to query #2 showing the “external” reference to the FHIR Resource Medication

The FHIR server was deployed as a Java servlet; this allowed us to set up a first set of tests using the web browser. Query type #1 (Table 2) requests the FHIR server to display FHIR DiagnosticReport resources, in which the diagnosis code is equal to “M321” in the ICD-10 coding system, corresponding to “systemic lupus erythematosus”. In this query, we also tested the “resource includes” mechanism to allow the client to request that the Patient and Encounter resources data be embedded in the results set in a special container, called “contained” (Fig. 7). Query type #2 requests the FHIR server to display FHIR MedicationOrder resources, in which the FHIR Medication identification code is equal to “3,400,893,219,874” in the CIP coding system, corresponding to “BISOPROLOL BGR 1.25MG CPR”. In this query, we also allowed the client to include data related to Patient, Encounter, and Medication FHIR resources (Fig. 8).

**Table 2:** Example of FHIR queries that can be executed using the i2b2 FHIR server

	FHIR query	Number of returned FHIR resources	Number of patients
#1	http://10.XXX.XXX.XX:8080/FhirServerV12/i2b2Layer/DiagnosticReport?code=M321&_include=DiagnosticReport:subject&_include=DiagnosticReport:encounter	80 FHIR DiagnosticReport	36
#2	http://10.XXX.XXX.XX:8080/FhirServerV12/i2b2Layer/MedicationOrder?medication.code=3400893219874&_include=MedicationOrder:patient&_include=MedicationOrder:encounter&_include=MedicationOrder:medication	503 FHIR MedicationOrder	290

Query type #1 and #2 displayed 80 FHIR DiagnosticReport resources for 36 distinct patients and 503 FHIR MedicationOrder resources for 290 distinct patients, respectively.

We validated the results displayed by the FHIR server in response to these two queries by asking the HEGP i2b2 database administrator to query the i2b2 database server to respond to the “client” requirements described in Figs. 1 and 2. The database administrator wrote two ad-hoc SQL queries for these two objectives and the results were extracted in csv files. All results (80 FHIR DiagnosticReport for 36 patients) displayed for query type #1 were manually verified and compared. For query type #2, we randomly chose 30 patients from the results displayed by the FHIR API and manually compared them to the corresponding results of the SQL query. The comparison showed exactly the same results.

## Discussion

The work reported here concerns the issue of data integration. Data integration is neither a recent research question [33] nor exclusively related to the healthcare domain [34]. Most current design and implementation solutions use a federated approach. The deployed solution leaves data at the source and provides querying access to the clients through a virtual federated view (schema) [35], also called virtual medical record (VMR) or virtual health record (VHR) in the medical informatics literature [36]. The VHR includes a CIM that defines generic concepts for representing healthcare data and a query language. The data client system poses queries against the VHR schema. The VHR should provide semantic and structural mapping between its concepts and those of the targeted data sources to respond to the needs of the clients. The advantage of this approach is the avoidance of the problem often referred to by the Arden Syntax community as the ‘curly braces problem’ [37], in which curly braces are used to isolate parts of code that are specific to the targeted health information system. This implies that developers of the site create two mapping stages to accommodate the data client system for its integration into their data source system: one between the client and the VHR and another between the VHR and the data source. In the federated approach, the mapping is designed and implemented only between the VHR CIM and the data source system if we assume that the data client system requests the data source system using the VHR query. Thus the data client system remains unaltered and its portability is facilitated [36].

Several articles have reported work in which mapping between a VHR CIM and a data source system (mostly an EHR) has been described [13]. The most often reported VHR CIM is the OpenEHR Archetype [13]. In a recent post [38], Thomas Beale, compared the FHIR and openEHR approach of clinical data modelling from a different point of view.

In terms of their aims, the objective of FHIR is to solve the problem of system-to-system communication, using the message approach, and the problem of system-to-application communication by allowing the development of APIs suited to programming. The objective of openEHR is to address the patient data challenge: i.e. creating long-lived, multi-version, distributed, and computable patient records that are future-proof and semantically enabled, using platform architecture based on formal semantics combined with terminologies, ontologies, drug databases service interfaces, etc.

In terms of their technical representation, the FHIR reference model is defined by a collection of information models, called resources, which can be profiled (or not) to generate a clinical information model. OpenEHR defines a reference model for the entire world and all openEHR data obey it. Various layers of models at the top, including archetypes, templates, and terminology subsets, allow the definition of a clinical information model. FHIR resources are produced from a superset of data found in legacy systems to be directly used by developers. In openEHR, models are generally built by communities of clinical domain experts, based on requirements.

In terms of platform coverage, openEHR and FHIR provide the same coverage level of patient information. The difference lies in the semantic models used. OpenEHR defines semantic querying using portable query language, AQL, whereas FHIR do not cover this aspect. However, FHIR coverage on APIs is substantially greater.

Studies in which mapping between a VHR CIM and a data source system has been reported covered a large variety of clinical domains, including nursing, oncology, neonatology, genetics, and infectious diseases [13]. Moreno-Conde et al. [13] analyzed these articles to assess whether there is an emergent consensus strategy for building and using CIM artefacts and whether it is possible to propose a common or unified CIM Process (CIMP). They described a CIM design and implementation process comprised of seven steps:

1. Scope definition leading to selection of the domain and selecting relevant experts
2. Analysis of the information covered in the specific domain
3. Design of the CIMs
4. Definition of implementable CIM specifications
5. Validation
6. Publishing and maintenance
7. Governance

In our approach, the first step corresponds to the identification and the formalization of the requirements of the “client”. The second step corresponds to the analysis of these requirements, which led to the identification of the various data elements targeted in both the i2b2 data model and structure and the FHIR resources and terminologies. We accomplished this by manually browsing the specifications of the FHIR resources. The flat structure of the FHIR CIM, represented by the UML notation, is clearly an advantage and the current descriptions made by the HL7 FHIR authors greatly facilitate browsing the model. For each FHIR resource, the authors describe the model using UML and several other formalisms. They describe the terminology which helps the developers determine whether it is necessary/recommended to map the data of the EHR with an existing coding system. They also list the possible parameters that can be used to implement FHIR Search operations.

Steps 3 and 4 correspond to the gap analysis that we have performed, between the data elements identified in the local context and FHIR resources, and the definition of our profile.

Step 5 concerns the manner in which the newly designed CIMs are validated. Various techniques have been adopted to validate the results of the previous steps, including peer review, the creation of prototype screens, and challenging the VHR CIM with routinely collected clinical data from multiple patients, which is probably the strongest [13]. We chose the latter technique, as one of the advantages of FHIR specification is the definition of an API specification to implement its CIM. This allowed us to explore and implement several mechanisms described in the FHIR API specifications and made available to developers through the Java HAPI FHIR API, which has proven to be a powerful tool. Searching for FHIR resources is probably the most common interaction that a FHIR server should perform for a client application. HAPI FHIR API allows implementation of this feature for two different FHIR resources: DiagnosisReport and MedicationOrder. For DiagnosisReport we were able to filter results using a “simple” search parameter: the DiagnosisReport code. This allowed us to filter the DiagnosisReport resources, resulting from the server, according to the value of a field that belongs to the sought FHIR resource. For the MedicationOrder resource, we were able to use a “chained” search parameter: FHIR Medication code. This allowed us to filter the FHIR MedicationOrder resources, resulting from the server, according to the value of a field that belongs to the FHIR resource referenced by the sought FHIR resource (Table 2). Resource referencing mechanisms allowed us to associate one or several resources to the main resource queried by the client in two different ways: internal “contained” references and external references. Figure 7 shows the case of referenced FHIR Resources (Patient and Encounter) packaged inside the source Resource (DiagnosticReport). Figure 8 shows the case of external references from the source Resource, MedicationOrder to the referenced resource, Medication, in which the Medication Resource is found at the end of the XML file returned by the server.

Step 6 concerns the publishing and maintenance of a profile. FHIR profiles are published in a FHIR registry called: Simplifier.net [39]. Our profile should be published soon in this registry.

### Limitations and upcoming improvements

Currently the i2b2 FHIR server supports only FHIR Search access operations to expose data related to two FHIR Resources: DiagnosticReport and MedicationOrder. We are implementing additional FHIR resources and plan to extend the server to other types of operations.

A terminology enrichment layer is currently under development. The implementation of this layer within the French context depends mainly on mapping efforts performed between the French and international coding systems. For medication, the French medication identification coding system is already mapped to ATC at the HEGP and has been used to enrich the FHIR server query results as depicted in Fig. 8. This mapping should be externalized for use in the implementation of the terminology enrichment layer.

For the other coding systems, the HEGP was a data provider in a large European project [10]. Two terminological mappings have been performed for a sub set of codes:

- An HEGP local coding system for health measurements and LOINC (Logical Observation Identifiers Names and Codes)
- A French coding system for clinical and surgical procedures, called classification commune des actes médicaux (CCAM) and SNOMED (The Systematized Nomenclature of Medicine).

We plan to use these mappings to enhance the terminology enrichment layer of the FHIR server.

Security is important when manipulating healthcare data. A security layer is under development. The server will include two levels of security recommended by the FHIR authors [40]:

- Authentication: to verify that the user is who they say they are. This layer will implement an HTTP basic Auth coupled with the local Lightweight Directory Access Protocol (LDAP) server.
- Authorization: to verify that the user is allowed to perform the given action. This layer will implement the AuthorizationInterceptor class of the HAPI FHIR API to examine the client request to determine whether a “write” or a “read” operation is legal.

Other aspects of security should be taken into consideration by developers when installing the server. For example, all clocks should be synchronized using NTP/SNTP and all exchange of production data should be performed using TLS/SSl, in addition to other measures [40].

The i2b2 FHIR server described in this article is based on FHIR DSTU2 version 1.0.0. An upgrade to the most recent FHIR version i.e. DSTU3 1.9.0 is currently under implementation. The most important change that can affect the i2b2 FHIR server is related to the introduction of a new interface called IBaseResource, from which the IResource interface extends. Many methods in the HAPI FHIR API which previously returned IResource now return IBaseResource. The HAPI FHIR authors describe how to handle these changes and others due to the migration from FHIR DSTU2 to FHIR DSTU3 [41].

Another important perspective of this work is its internal validation by testing the server with a full-FHIR clinical decision support system dedicated to verifying medication prescriptions for the elderly. External validation will be performed by testing the server with a Harvard SMART app [20].

## Conclusion

We have been able to show the feasibility of implementing a FHIR layer over the i2b2 database model to expose data of the CDW as a set of FHIR resources using the HAPI FHIR API. Our i2b2 FHIR server could be easily used/adapted by other developers to expose their local data in FHIR format. The DAO layer implemented is suitable for other i2b2 users and only requires adaptation of the service class package, where HQL queries should be rewritten according to the local i2b2 data storage model and/or according to other requirements. The FHIR resource provider classes should also be adapted, as these classes handle the mapping between the i2b2 model and the FHIR resources exposed by the server. The code source is freely available online as open source.

## Availability and requirements

- **Project home page:** http://im-ageproject.weebly.com
- **Operating system:** cross-platform
- **Other requirements:** Java Eclipse with plugins for: Git and Maven.
- **License:** MIT License
- **Download:** https://github.com/3abdel3ali/HAPI-FHIR-based-I2B2-Layer

## Abbreviations

API: Application Programming Interface
ATC: Anatomical Therapeutic Chemical
CDW: Clinical Data Warehouse
CIM: Clinical Information Model
CIP: Code Identifiant de Présentation (French Medication Code Id)
CIS: Clinical Information system
DAO: Data Access Objects
EHR: Electronic Health Record
FHIR: Fast Healthcare Interoperability Resources
HEGP: Hôpital Européen Georges Pompidou (Georges Pompidou European Hospital)
HL7: Health Level seven
ICD-10: International Statistical Classification of Diseases and Related Health Problems
LOINC: Logical Observation Identifiers Names and Codes
OID: Objects Identifier
RIM: Reference Information Model
SNOMED: The Systematized Nomenclature of Medicine
UML: Unified Modeling Language
